# Single or double headed capsules for the investigation of suspected small bowel bleeding: Are two heads better than one

**DOI:** 10.3389/fgstr.2022.1071797

**Published:** 2022-12-16

**Authors:** Eilis McCarthy, Sandeep Sihag, Charlene Deane, Caroline Walker, Serhiy Semenov, Barbara Ryan, Niall Breslin, Anthony O’Connor, Sarah O’Donnell, Deirdre McNamara

**Affiliations:** ^1^ Department of Gastroenterology, Tallaght University Hospital, Dublin, Ireland; ^2^ Trinity Academic Gastroenterology Group, Research Centre, School of Medicine, Trinity College, Dublin, Ireland

**Keywords:** video capsule endoscopy, suspected small bowel bleeding, single or double headed capsule, small bowel access, GI bleeding

## Abstract

**Background:**

Capsule endoscopy is now the accepted first line investigation for suspected small bowel (SB) bleeding. Recent evidence suggests the diagnostic yield for SB pathology may be higher for tailored double headed (DH) SB capsules. Whether other forms of bidirectional capsules offer a similar advantage is less clear.

**Aim:**

To compare the efficacy of single headed versus bidirectional capsules in detecting pathology in patients with suspected small bowel bleeding.

**Methods:**

A single centre prospective comparison study was conducted over an 8 month period in a tertiary care hospital. Patients referred with overt or suspected SB bleeding were assigned to either SB3 Medtronic SB capsule (SH) during the initial four months or PillCam Colon 2 Medtronic capsule (DH) during the subsequent four months. Studies were analysed by trained Capsule Endoscopists and approved by our institutions capsule review board. Findings were compared between SH and DH capsules using a chi^2^ or t-test as appropriate. A p value of <0.05 was considered significant.

**Results:**

201 subjects were included, mean age 61.8 years, 90 (45%) male. Majority referred with occult bleeding, 153 (76%). DH and SH capsule used in 100 and 101 cases, respectively. 90% (n=181) capsules were complete and overall diagnostic yield was 57% (n=114). Diagnostic yield was similar between both groups - DH 53% (n=53), SH 60% (n=61). Positive finding in overt bleeding; SH 85% (n=22) versus DH 50% (n=11), p<0.02. SH capsules more frequently detected SB inflammation, 27 (27%) versus 9 (9%), p<0.002. More patients had another diagnosis in the DH (19) than the SH (9), p<0.04, the majority were type 1a vascular lesions, “red spots” or diminutive colonic polyps.

**Conclusion:**

Single head and double head capsules perform similary in terms of diagnostic yield overall. This supports the continued use of standard small bowel capsules for investigation of the small bowel.

## Introduction

Small bowel capsule endoscopy is the universally accepted first line investigation for patients with suspected small bowel bleeding (1–3). In addition, colon capsule endoscopy (CCE) is now a recommended alternative means to assess the colonic mucosa in a variety of clinical settings (4, 5). The use of capsules with extended battery life, and usually two bidirectional cameras, as a means of pan-intestinal examination of the gastrointestinal tract has also been explored. A recent systematic review confirmed, pan-intestinal capsule studies with either CCE capsules or specifically designed pan-intestinal capsules (PCE) is a feasible technique, although the majority of studies included were undertaken in an IBD cohort rather than for bleeding (6). More recently, an Italian study confirmed pan-intestinal assessment using CCE (PillCam Colon 2, Medtronic Inc, Dublin, Ireland) was an effective investigation strategy in patients with melena (7). In this study, small bowel bleeding and interventions were undertaken in 50% of cases, suggesting double headed (DH) CCE capsules are a sensitive test for small bowel bleeding. Similarly, a recent multicentre UK study reported early success with the use of a specifically designed bidirectional DH small bowel capsule (MiroCam® MC2000, Synectics Med LTD, Enfield, UK) for patients with suspected small bowel bleeding (8). In this study, 29% of examinations reported differences in single headed and double headed readings, of which 18% were clinically significant. They went on to suggest the routine adoption of DH capsule endoscopy for small bowel assessment should be considered. The potential advantages of a bidirectional, DH capsule include a wider field of view and usually a longer battery life, and in some an adapted frame to allow for better image capture during periods of rapid transit. However not all capsules are equal and lesion detection may also be affected by other technical issues including image resolution, dynamic range imaging and other specifications specific to one type of capsule. For most centres, ideally a single capsule, which has the potential to be used for small bowel, colon and pan-intestinal assessments, would be advantageous rather than stocking a larger number of site or indication specific capsule options. Based on current data Pillcam colon 2 capsules may offer just that opportunity to centres and warrants further investigation. We therefore conducted a prospective comparison study to compare the efficacy of single headed small bowel SB3 capsules with bidirectional double headed PillCam Colon 2 capsules for the detection of small bowel pathology in patients with suspected small bowel bleeding.

## Materials and methods

Following ethical approval adult patients referred for an index capsule endoscopy with suspected small bowel bleeding, either overt or occult, with negative bidirectional endoscopy, were sequentially assigned to have a small bowel study with either a SH SB3 capsule or DH Pillcam Colon 2 capsule (CCE) (Medtronic Inc, Dublin, Ireland). A SB3 capsule was used in all patients in the first 4 months of the study, and a CCE capsule in all patients in the following 4 months. Standard capsule exclusion criteria were applied including; dysphagia, known strictures, failed patency test and pregnancy. In addition, patients with known Crohn’s or other small bowel pathology, altered gastric or small bowel anatomy, endoscopic capsule placement, previous capsule studies and previous device assisted enteroscopy with intervention were also excluded. All patients followed the same standard pre-procedure protocol including discontinuation of NSAID for 4 weeks. For DH studies, the sleep mode was manually turned off prior to ingestion, to allow for complete small bowel imaging. As per our unit protocol, patients at risk of delayed gastric transit underwent routine assessment of gastric passage with administration of 10mg of Metoclopramide if not confirmed. In addition, all patients received Simethicone 125mg PO, (Windeeze, Perrigo, Devon, UK) at least 30 minutes prior to ingestion. All studies were analyzed by trained Capsule Endoscopists using Rapid Reader software version 9.0 and findings were approved by our institutions capsule review board. Standard descriptions for vascular and inflammatory lesions were applied (9, 10). Significant enteritis was defined as > 3 ulcers and/or a Lewis score of > 135 (11, 12).

Patient demographics, capsule findings and outcomes were recorded and compared between groups using a chi^2^ or t-test as appropriate. A p value of <0.05 was considered significant.

## Results

In total 201 patients were enrolled, 100 PillCam Colon 2 and 101 SB3 cases. Patient demographics were similar in both groups. The indication for capsule was overt bleeding in 24% (n=48) and occult bleeding in 76% (n=153) and was similar in both groups, DH 22% and SH 26%. In all 90% (n=181) of studies were complete, image quality was good/adequate in 95% (n=191). The main outcome, overall diagnostic yield was 57% (n=114). Findings included angiodysplasia in 29% (n=33), enteritis (NSAID or Crohn’s) in 18% (n=36), active bleeding without an obvious source in 8% (16), other findings in 28% (25%) and 1 patient (1%) had a small bowel tumor. Diagnoses included in the “other” category included findings of enteropathy (coeliac disease), polyps, non-specific enteritis, Meckel’s diverticulum and colonic findings (**Table 1**).

**Table 1:** Study Population overall and by group.

	Total N (%)	DH N=100	SH N=101
**Male**	90 (45)	38	52
**Female**	111 (55)	62	49
**Mean Age (years)**	61.8 (16-94)	61.8	61.9
**Indication**			
**Overt Bleeding**	48 (24)	22	26
**Occult Bleeding**	153 (76)	78	75
**Complete study**	181 (90)	88	93
**Incomplete study**	20 (10)	12	8
**Positive Study**	114 (57%)	52	59
**Findings**			
**Angiodysplasia**	33 (29)	15	18
Enteritis*	36 (18)	9	27
**Active Bleeding**	16 (8.0)	12	4
**Tumour**	1 (1.0)	0	1
Other*	28 (25)	19	9

*Enteritis defined as positive Lewis >135/> 3 ulcers.

*Other category included findings of enteropathy, polyps, non-specific enteritis, Meckel’s diverticulae, colon findings.

There was no difference in basic patient demographics (age or gender) between the two groups. Both completion rates and small bowel transit times were also similar. There was no difference in the overall diagnostic yield for DH and SH capsules, 53% (n=53) versus 60% (n=61). More patients in the SH group with overt bleeding had a positive finding, 85% (n=22) versus 50% (n=11), p<0.02, 95%CI 9.54-59.7. Overall a similar number of patients were found to have vascular lesions or active bleeding, SH 22 (22%) and DH 27 (27%), although a greater proportion of patients in the DH group had active bleeding without an identifiable source, 44% (n=12) versus SH 18% (n=4), p<0.04. Significant enteritis was more frequently detected by SH capsules, 27% (n=27) versus DH capsules, 9% (n=9), p<0.002. More patients had another diagnosis in the DH (19) than the SH (9), p<0.04, the majority were type 1a vascular lesions, “red spots” (7) or diminutive colonic polyps (6) (**Table 2**).

**Table 2:** Comparison of single headed (SB3) and double headed (PillCam Colon 2) capsule performance.

	Single Headed n=101	Double Headed n=100	Significance
**Completion Rates %**	92	88	NS
**Small Bowel Transit**	247 (minutes)	234 (minutes)	NS
**Poor Image Quality %**	4	5	NS
**Diagnostic Yield**			
Overt Bleeding	85% (n=22)	50% (n=11)	P<0.02
Occult Bleeding	52% (39)	54% (32)	NS
**Findings**			
Enteritis	27	9	P<0.002
Bleeding no source	4	12	P<0.04
Angiodysplasia	18	15	NS
Tumour	1	0	NS
Other	9	19	P<0.04

*NS, not significant, p value >0.05.

## Discussion

Our prospective study compares the performance of SB3 and PillCam Colon 2 capsules for the investigation of suspected small bowel bleeding. Previous studies of pan-intestinal capsule investigation and double headed small bowel capsules have suggested a bidirectional, double headed, capsule, including PillCam Colon 2, designed for colonic imaging, may have a diagnostic advantage in the small bowel (6, 7, 13, 14). Our findings do not support this hypothesis.

One such study (13), determined the use of DH was feasible and suggested the use of two heads may increase diagnostic yield, however this was a non-comparative study and could not hypothesize whether DH would out-perform SH capsules. A further study (14) suggested forward-facing orientation of the capsule when entering the SB improves diagnostic yield when using a SH camera, and potential missed diagnoses due to inability to control the orientation could be corrected by using a DH capsule. Our results do not support this. General performance measures, completion rate, image quality, transit times and overall diagnostic yield were similar between the two capsules.

Interestingly; small bowel, single headed, SB3 capsules had a higher diagnostic yield in patients with overt bleeding, 85% versus 50% and the SB3 capsule was more sensitive for significant enteritis 27% versus 9%. This is likely due to technical differences between the two, with the SH SB3 being specifically tailored to the small bowel. While there is ongoing debate as to the long-term clinical relevance of enteritis as defined by either the Lewis score or Mow criteria, they remain the best way to differentiate significance (9, 10). Of note, the mean LS in positive patients was 354.3 +/- 76, and was similar in both groups, and no patients were diagnosed with significant enteritis with only 4 ulcers. This unsurprisingly suggests using a capsule produced specifically for the small bowel is advantageous for detecting small bowel pathology. PillCam Colon 2 had a higher yield for “other” diagnoses, including colon, which is to be expected, and if excluded would worsen the comparison with SB3 yield further. In addition, of those with a positive study, more in the DH group had bleeding without an identifiable source, which is not likely to be relevant as the overall yield was lower than for SB3 patients with overt bleeding and a definitive diagnosis.

The sequential design of our study, whereby one and then the other capsule was used for a period of time rather than the random allocation of patients to either at the same time could appear to introduce bias. However, both study populations were similar and the overall diagnostic yield adequate, and within expected parameters. The reason for the design was to simplify the process for our technicians, to ensure the sleep mode was routinely turned off during small bowel studies with a PillCam Colon 2 capsule. In addition, the Readers would not have been blinded by either design, as to the type of capsule used, simply because the software display is inherently different.

Of note, we did not compare the performance of single camera to double camera reading of our PillCam Colon 2 studies, because we believed the yield would logically be better using both cameras, as proven previously (6). This would also not have helped us to determine if a double head, non-small bowel specific capsule, was superior to a single headed small bowel one.

Despite the relatively small study population and design issues, our study at least suggests SB3, a single headed capsule is not inferior to a double head, PillCam Colon 2 capsule, which also have a longer battery life and adaptive frame rate technology, for small bowel assessment, and that other design specifications are as important. While there are potential advantages to a service to having a single capsule for all capsule indications, we suggest further studies would be required including cost benefit analysis before the routine use of double headed capsules could be recommended.

## Conclusion

There was no difference in the overall diagnostic yield for double-headed (PillCam Colon 2) and single-headed (SB3) capsules in patients presenting with suspected small bowel bleeding. Our findings support the ongoing use of specifically designed small bowel capsules for the assessment of small bowel disease.

## Data availability statement

The original contributions presented in the study are included in the article/supplementary material. Further inquiries can be directed to the corresponding author.

## Ethics statement

The studies involving human participants were reviewed and approved by Taillight University Hospital Ethics Committee. The patients/participants provided their written informed consent to participate in this study.

## Author contributions

EM – first author, assisted with data collection, data analysis and drafting the manuscript SS, CD, CW, SS assisted with data collection and contributed equally to this work BR, NB, AO, SO participated in design of the study and contributed equally to this work DM – senior and last author, designed and oversaw the study, performed statistical analysis and drafted the manuscript. All authors contributed to the article and approved the submitted version.
